# Intra- and Interseasonal Autoregressive Prediction of Dengue Outbreaks Using Local Weather and Regional Climate for a Tropical Environment in Colombia

**DOI:** 10.4269/ajtmh.13-0303

**Published:** 2014-09-03

**Authors:** Matthew D. Eastin, Eric Delmelle, Irene Casas, Joshua Wexler, Cameron Self

**Affiliations:** Department of Geography and Earth Sciences, University of North Carolina, Charlotte, North Carolina; Department of Social Sciences, Louisiana Tech University, Ruston, Louisiana

## Abstract

Dengue fever transmission results from complex interactions between the virus, human hosts, and mosquito vectors—all of which are influenced by environmental factors. Predictive models of dengue incidence rate, based on local weather and regional climate parameters, could benefit disease mitigation efforts. Time series of epidemiological and meteorological data for the urban environment of Cali, Colombia are analyzed from January of 2000 to December of 2011. Significant dengue outbreaks generally occur during warm-dry periods with extreme daily temperatures confined between 18°C and 32°C—the optimal range for mosquito survival and viral transmission. Two environment-based, multivariate, autoregressive forecast models are developed that allow dengue outbreaks to be anticipated from 2 weeks to 6 months in advance. These models have the potential to enhance existing dengue early warning systems, ultimately supporting public health decisions on the timing and scale of vector control efforts.

## Introduction

Severe outbreaks of vector-borne infectious diseases and their likely geographic expansion pose a serious threat to vulnerable populations. Over 2.5 billion people living in tropical and subtropical climates are at potential risk of contracting dengue fever, which has become the most important vector-borne viral disease in the world.^1^ Current estimates suggest that up to 100 million dengue cases occur annually, of which 1 million cases require hospitalization for the most serious form: severe dengue (formerly known as dengue hemorrhagic fever). Across Latin America, despite largely successful efforts in the 1940s to eradicate the primary dengue fever vector—the female *Aedes aegypti* mosquito—from most countries,^2^ dengue has gradually re-emerged across the continent because of several factors, including re-emergence of the vector, population growth, increased global travel, diversification of viral strains, and insecticide resistance.^3^,^4^ Rapidly growing urban areas are most vulnerable because of poorly planned urbanizations and migrations.

Classic dengue fever is distinguished by headache, fever, eye pain, sore muscles and joints, nausea and vomiting, and swollen glands, and it mainly affects children and younger adults.^5^ Severe dengue causes internal hemorrhaging and significant plasma loss that can lead to dengue shock syndrome, which has fatality rates nearly 50 times higher than that of classic dengue fever. Because no licensed vaccine exists, controlling or preventing dengue outbreaks is the only alternative, which is implemented by reducing its primary vector through monitoring and habitat control programs. If uncontrolled, outbreaks in urban areas can impact nearly 80% of the population^6^,^7^ and incur over $100 million in mitigation and treatment expenses^8^; the potential social and economic costs of dengue are comparable with the costs of malaria.^9^ As a result, effective low-cost early warning systems (EWSs) capable of predicting potential dengue outbreaks in a timely manner are critical to enhance decision-making.

### Background.

Numerous studies have documented the life stages of *Ae. aegypti*, including its preferred habitats, range, behavior, and sensitivity to local environmental conditions. The mosquito is peridomestic, is day-biting, and feeds almost exclusively on human blood.^10^,^11^ Its breeding habitat consists of stagnant pools from man-made cisterns to sewers and discarded tires.^12^,^13^ The *Aedes* development, behavior, and survival are temperature- and humidity-dependent, whereas the presence of water is necessary for egg laying and larval development.^14^–^16^ Ambient temperatures between roughly 5°C and 40°C are required for mosquito survival, but greater mosquito densities occur when temperatures remain within 15–32°C.^17^–^22^ Adult mosquitoes will only feed when ambient temperatures exceed 18°C,^23^ and feeding frequency increases when temperatures are warm and humidity is low.^24^ Finally, although it is generally recognized that greater mosquito densities are expected during and after a rainy season, the effect of precipitation seems to be site specific—dependent on not only rainfall accumulation, frequency, and intensity^25^–^27^ but also, variability in the number of man-made breeding habitats to retain water during periods of less rainfall.^28^,^29^

Prior studies have also explored the sensitivity of the dengue virus and its transmission dynamics to changes in local environmental conditions. Consensus suggests that viral development (or the extrinsic incubation period inside the vector) and transmission (a function of mosquito density, feeding frequency, and survival) occur more rapidly and more frequently at warmer temperatures,^16^,^30^ with peak transmission occurring when mean temperatures are confined between 27°C and 30°C.^20^ Likewise, virus amplification (survival) at temperatures below 18°C (12°C) is rare.^18^,^31^ A recent study suggested that dengue transmission is dependent on daily temperature and temperature range; when mean temperatures exceed 18°C, larger diurnal temperature ranges decrease transmission, presumably because of a reduction in vector survival and viral amplification during the cooler hours of each day.^32^

A limited number of recent studies have developed site-specific multivariate regression models using various combinations of time-lagged weather parameters to predict local dengue incidence and/or mosquito density in Australia,^21^ Taiwan,^33^ Singapore,^27^,^34^,^35^ New Caledonia,^36^ Mexico,^37^,^38^ Guadeloupe,^39^ and Puerto Rico.^13^ The best-fit model from each study was deemed skillful, but no common set of optimal weather-based predictors or time lags has been identified among the studies, suggesting that effective EWSs require careful consideration of local (e.g., city or province scale) environmental factors.

### Objectives.

The objectives of this study are to (1) improve our knowledge of the relationships between environmental factors and dengue fever transmission on biweekly to seasonal time scales and (2) develop multivariate intra- and interseasonal predictive models of dengue incidence that can enhance an EWS for Cali, Colombia. To this end, we use 12 years (2000–2011) of epidemiological and meteorological data to identify significant time-lag relationships between reported dengue cases and local weather or regional climate predictors. We recognize that dengue transmission is a function of complex relationships between hosts, vectors, and their environment,^40^ but if related weather/climate parameters can be used to predict periods of elevated dengue risk, then communities could implement cost-effective mitigation strategies to control the dengue outbreaks in a timely manner.^34^,^35^

## Materials and Methods

### Study area.

**Figure 1.**
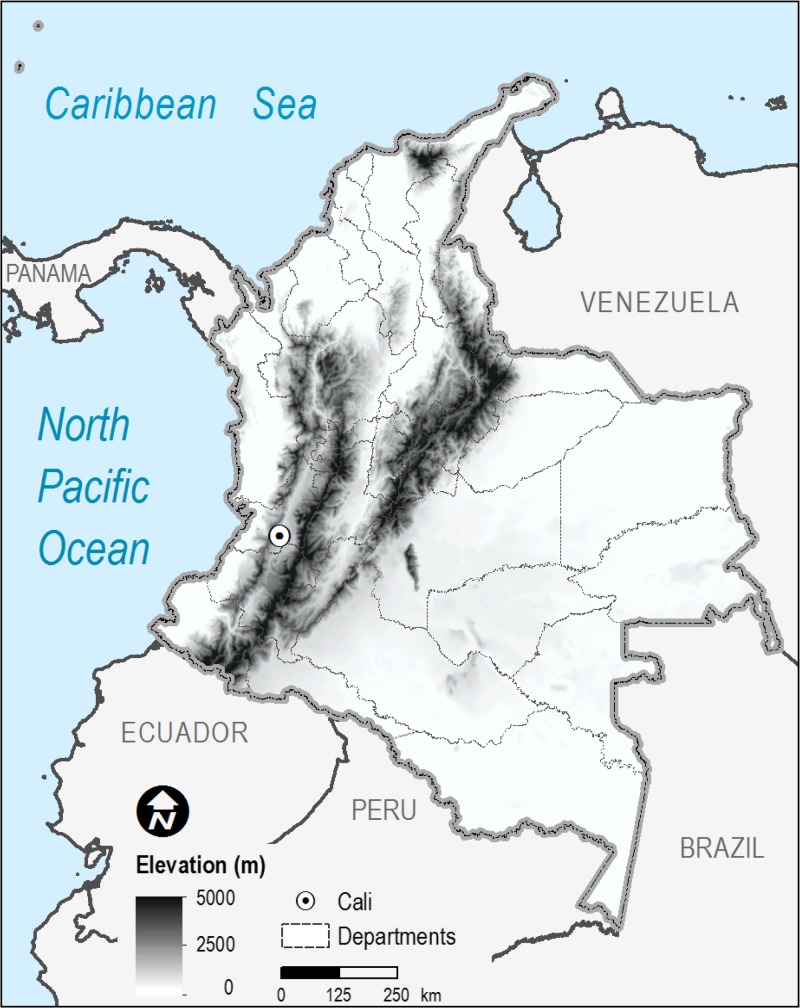
Topographical variation across Colombia, South America. The city of Cali is located within the tropical climate of the Cauca River Valley between two mountain ranges with peaks in excess of 4,000 m.

Tropical and subtropical urban environments that have experienced rapid unorganized growth are particularly vulnerable to dengue fever outbreaks. One such dynamic urban environment is the city of Cali, Colombia (Figure 1), which is the third largest metropolitan area in the country with an estimated 2010 population of 2.3 million spread across 564 km^2^ (with a population density over 4,000/km^2^). The city (at 3.42° N, 76.52° W and 997 m above sea level) is located within the Cauca River Valley between two mountain ranges with peaks in excess of 4,000 m, but it has a tropical climate with two distinct warm-dry seasons (December to February and June to September; mean maximum temperature = 31.9°C; mean monthly rainfall = 54.6 mm) and two cooler-wet seasons (March to May and October to November; mean maximum temperature = 28.0°C; mean monthly rainfall = 107.4 mm). Of critical concern are peripheral neighborhoods, which have experienced a massive influx of migrants. These neighborhoods are characterized by a high-density, low-income population that suffers from unplanned urbanization, including squatter settlements along the river banks.^41^ Such regions contain limited sewer infrastructure with several open-air waste water channels, and many households rely on rainfall cisterns for drinking water. Thus, ample sources of stagnant water (i.e., ideal mosquito-breeding habitats) are present around the city.

### Epidemiological data.

The database used in this study corresponds to the dengue fever cases reported in the Sistema de Vigilancia en Salud Pública (SIVIGILA; English—Public Health Surveillance System) for the city of Cali from 2001 to 2011. Information for each dengue case includes patient information (e.g., sex, age, race, and neighborhood), diagnosis date, first symptoms date, hospitalization date (if any), final condition, and reporting institution. All cases are confirmed in the laboratory using standard methods (by a complete hemogram and immunoglobulin M [IgM] test). Here, to explore the temporal relationships between dengue cases and variations in local weather and/or climate, we use the diagnosis date. Intracity spatial patterns of the reported cases are discussed in detail elsewhere.^42^,^43^

**Figure 2.**
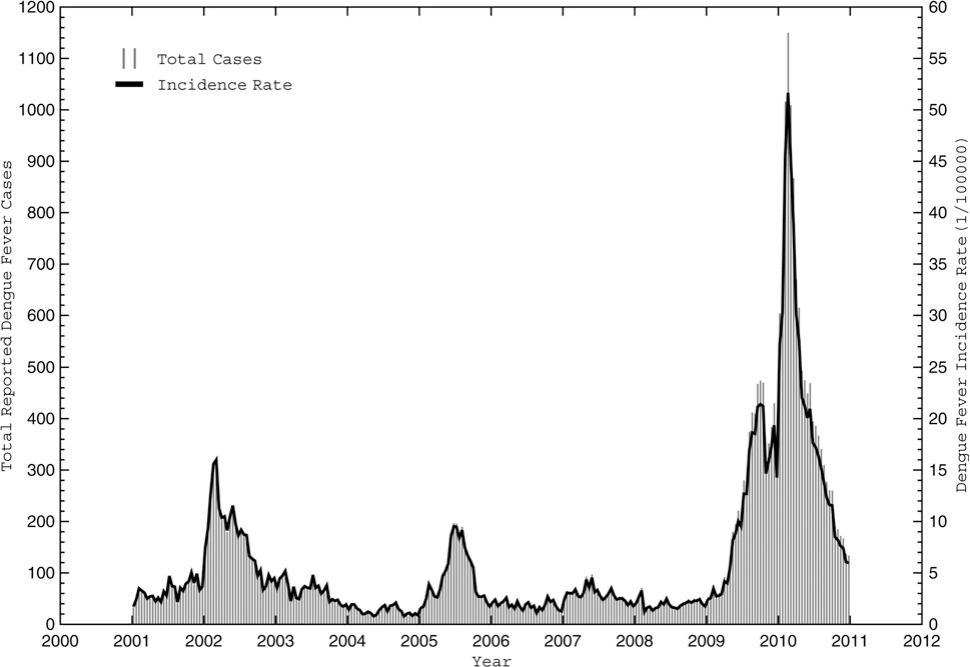
Reported biweekly dengue case totals and population-adjusted incidence rates from 2001 to 2010. Biweekly case totals (D_TOT_) are shown by vertical gray lines, and the population-adjusted incidence rates (D_POP_) are depicted in black for Cali during the model development period between January of 2001 and December of 2010.

In total, 34,970 dengue fever cases were reported in Cali between January of 2001 and December of 2011, accounting for 6% of the total cases reported in Colombia during the same period. Re-emergence of dengue fever was first reported in the Cauca River Valley in the 1970s, and the first cases of severe dengue fever occurred in 1989.^44^ Since that time, large-scale outbreaks swept across the valley in 1991, 1994–1995, 1998, 2001–2002, 2005, and 2009–2010; the latter three (included in our database) (Figure 2) resulted in over 4,000, 2,500, and 15,000 reported cases, respectively, within Cali alone.

With SIVIGILA in place since the 1960s, it provides a robust and consistent measure of dengue cases across the study area. However, a couple of factors must be addressed before performing a long-term temporal analysis and prediction. First, because of a variety of societal factors that could introduce bias or artificial peaks in the daily dengue counts (e.g., holidays, school/work schedules, and operating hours at clinics), we computed biweekly and monthly dengue counts from the daily database. Second, because of rapid population growth in Cali (35.4% between 1993 and 2011; from 1,641,698 to 2,230,000), dengue counts were standardized by population assuming steady linear growth during the study period. Third, we used 2001–2010 counts for development of the predictive models (hereafter referred to as the developmental data) and set aside 2011 counts for independent verification of the predictions (hereafter referred to as the verification data). Shown in Figure 2 are the total reported dengue cases (D_TOT_) and the biweekly population-adjusted dengue incidence rate (D_POP_) reported in Cali from 2001 to 2010.

### Meteorological data.

Daily weather observations recorded at the Cali international airport between January of 2000 and December of 2011 were obtained from the quality-controlled Global Historical Climate Network (GHCN) archive^45^,^46^ maintained at the National Climate Data Center (NCDC; http://www.ncdc.noaa.gov/). Any missing GHCN data (e.g., numerous rainfall observations during 2008 and 2009) were supplemented with raw observations obtained from the Colombian Institute of Hydrology, Meteorology and Environmental Studies; despite dissimilar quality control procedures, regular duplicate records among the two datasets provide confidence in our simple replacement of missing GHCN data with raw observations.

**Figure 3.**
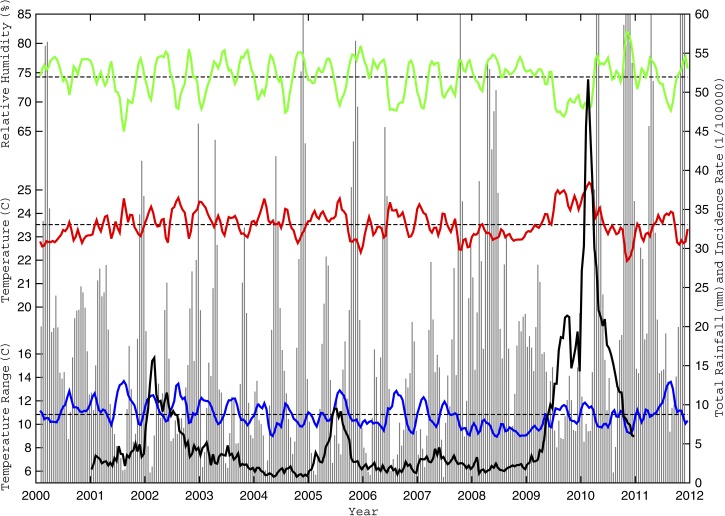
Observed biweekly weather variability and reported dengue incidence rates from 2000 to 2011. The biweekly mean temperature (T_AVG_), mean relative humidity (RH_AVG_), and mean daily temperature range (ΔT_AVG_) are shown in solid black, total rainfall (RR_TOT_) is shown by vertical gray lines, and the population-adjusted dengue fever incidence rate (D_POP_) is shown in thick dashed black for Cali from January of 2000 to December of 2012. The thin dashed black horizontal lines denote the T_AVG_, ΔT_AVG_, and RH_AVG_ long-term baseline means. Only dengue data during the model development period (2001–2010) are shown.

To be consistent with the dengue case data, several biweekly and monthly weather parameters were computed from the daily observations for statistical analysis: mean temperature (T_AVG_), maximum temperature (T_MAX_), number of days with T_MAX_ greater than 32°C (G32_DAYS_), minimum temperature (T_MIN_), number of days with T_MIN_ less than 18°C (L18_DAYS_), mean daily temperature range (ΔT_AVG_), maximum daily temperature range (ΔT_MAX_), mean relative humidity (RH_AVG_), relative humidity range (ΔRH), total accumulated rainfall (RR_TOT_), and the number of days with measurable rainfall (RR_DAYS_) during each time period (Table 1). Parameter selection was motivated by the aforementioned studies addressing *Ae. aegypti* sensitivity to their environmental conditions. However, two previously unevaluated parameters (ΔT_AVG_ and RR_DAYS_) were tested to determine if large daily temperature ranges (which might reduce outdoor mosquito survivability but also encourage more mosquitoes to seek a less variable environment indoors) were more relevant than daily extreme temperatures and if regular rainfall (which maintains regular sources of stagnant water for breeding) was more relevant than total rainfall, respectively. Figures 3

–5

**Figure 4.**
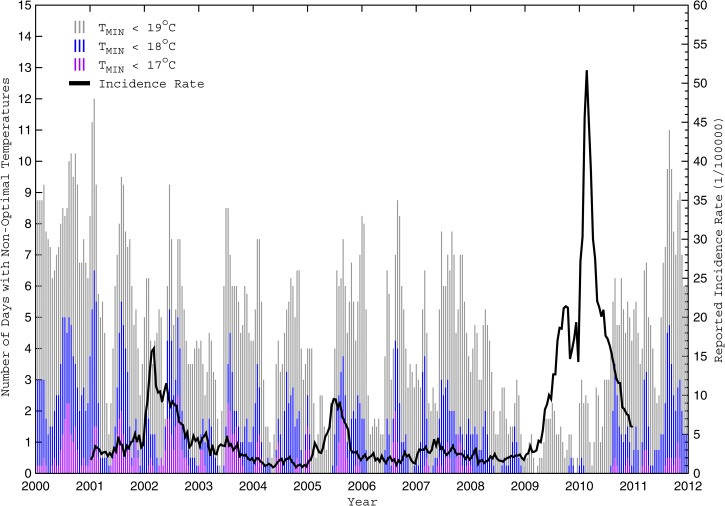
Number of days with non-optimal cold temperatures and reported biweekly dengue incidence rates from 2000 to 2011. The numbers of days during each biweekly period with a minimum temperature (T_MIN_) less than 19°C, 18°C, and 17°C are depicted by vertical light grey, medium grey, and dark grey lines, respectively, for Cali from January of 2000 to December of 2011. Note that a simple 1–2–1 filter was applied to the non-optimal daily counts for greater clarity. Also shown in black is the biweekly population-adjusted dengue fever incidence rate (D_POP_) from 2001 to 2010.

show the biweekly D_POP_ with a select set of biweekly weather parameters for the entire study period (2000–2012).

**Figure 5.**
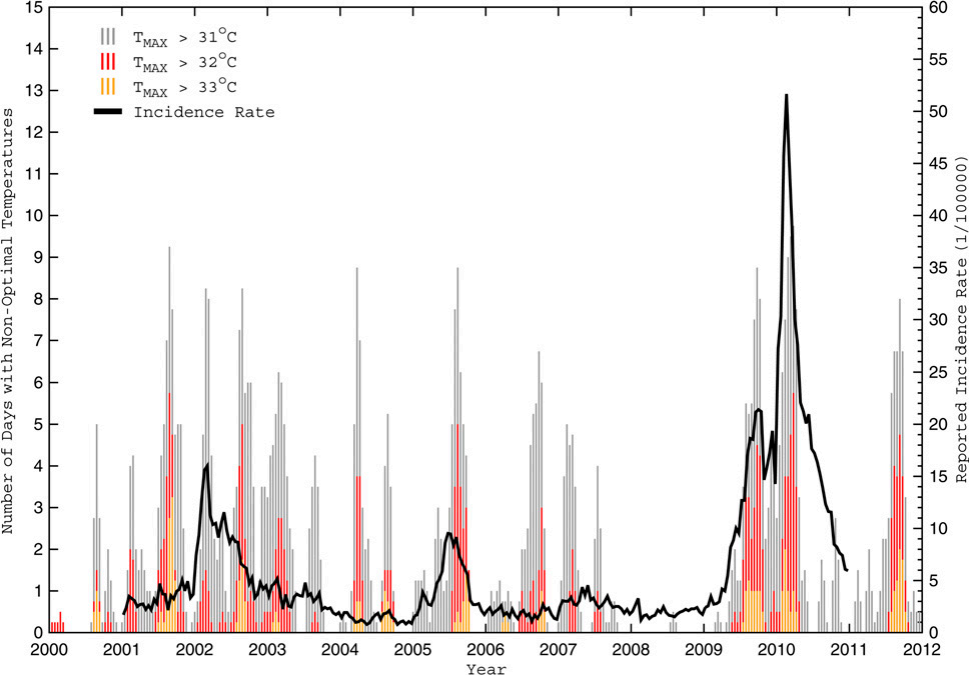
Number of days with non-optimal warm temperatures and reported biweekly dengue incidence rates from 2000 to 2011. The numbers of days during each biweekly period with a maximum temperature (T_MAX_) greater than 31°C, 32°C, and 33°C are depicted by vertical grey, red, and orange lines, respectively, for Cali from January of 2000 to December of 2011. Note that a simple 1–2–1 filter was applied to the non-optimal daily counts for greater clarity. Also shown in black is the biweekly population-adjusted dengue fever incidence rate (D_POP_) from 2001 to 2010.

**Figure 6.**
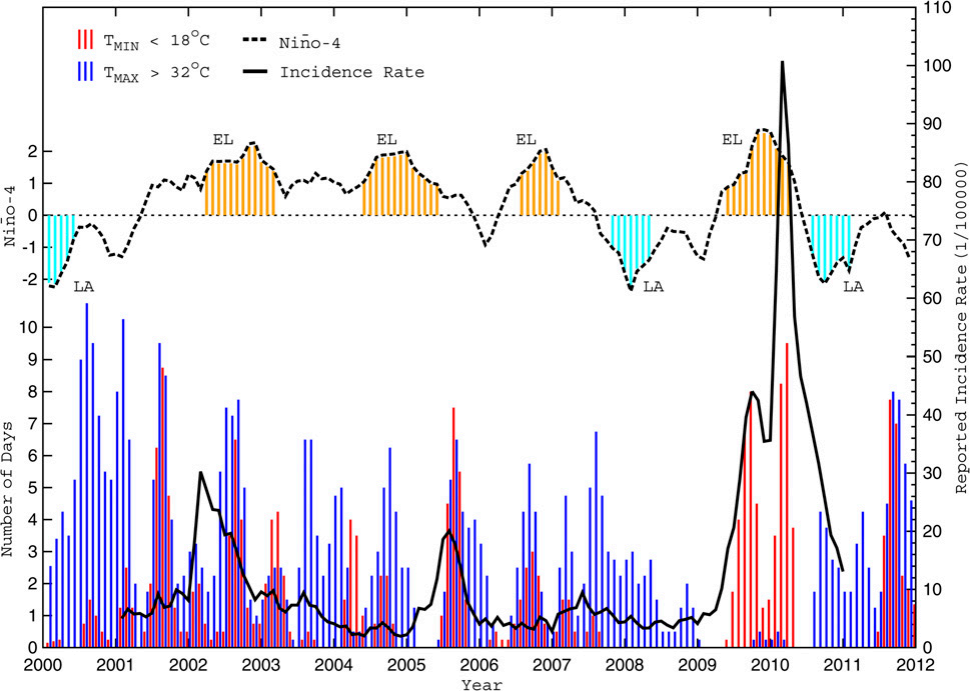
Monthly time series of non-optimal days, ENSO, and dengue incidence rate from 2000 to 2011. The number of days each month with a maximum temperature (T_MAX_) greater than 32°C is shown by vertical light grey lines, whereas the number of days with a minimum temperature (T_MIN_) less than 18°C is depicted by vertical bark grey lines for Cali from January of 2000 to December of 2011. Note that a simple 1–2–1 filter was applied to the non-optimal daily counts for greater clarity. Also shown is population-adjusted dengue fever incidence rate (D_POP_; solid black) and normalized N_4_ index (dashed black), where vertical dotted grey lines denote significant El Niño (EL) and La Niña (LA) events.^47^

Analysis of climatic relationships with Cali's dengue incidence rates was restricted to the El Niño Southern Oscillation (ENSO) because of its close proximity. The state of ENSO during our study period was determined from the monthly Southern Oscillation Index (SOI) as well as the monthly Niño-1.2 (N_12_), Niño-3 (N_3_), Niño-4 (N_4_), and Niño-3.4 (N_34_) indices (Table 1) readily available from the Climate Prediction Center (CPC; http://www.cpc.ncep.noaa.gov/). Using a standard definition, significant El Niño (La Niña) events occur when the 5-month running mean of sea surface temperature (SST) anomalies (relative to the 1971–2000 base period) for a given index exceeds +0.4°C (−0.4°C) for at least 6 consecutive months.^47^ Based on the N_4_ index (Figure 6), a total of four El Niño events and two La Niña events occurred during our study period.

### Statistical analysis.

Overall, the biweekly and monthly D_POP_ values were compared with the full set of computed weather parameters and climate indices (listed in Table 1) across a range of lags and then predicted using an optimal form of an autoregressive integrated moving average (ARIMA) model with transfer functions. Other multivariate regression models (linear, Poisson, and negative binomial) were evaluated with a subset of our developmental data,^48^ but the ARIMA transfer models produced the best fit, which is in agreement with previous studies.^49^

Our model development methods were as follows. First, any non-normal distributions in the developmental dataset (2000–2010) were dealt with through transformations; the D_POP_, G32_DAYS_, L18_DAYS_, and RR_TOT_ parameters were transformed using log_10_(*x* + 1) to meet the requirements of regression analysis. Second, normalized anomalies from their respective long-term mean during the period were computed for D_POP_ and each meteorological parameter. Third, each normalized meteorological time series was checked for statistically significant long-term trends (none were identified at the 5% level) and then pre-whitened to account for any non-randomness or serial correlation. Fourth, through examination of the D_POP_ autocorrelation and partial correlation functions, we progressively adopted ARIMA form until the residuals exhibited temporal randomness and normality (or an appropriate predictive form). Fifth, cross-correlations between each pre-whitened normalized parameter and D_POP_ over a range of biologically plausible (with respect to the *Aedes* lifespan) time lags (*t* = 0 to −8) were used to select the most significant independent variables among each meteorological group (temperature, temperature range, humidity, rainfall, and climate) (Table 1). Sixth, all possible combinations of the most significant independent lagged parameters were incorporated into the appropriate ARIMA models using transfer functions and then evaluated for goodness of fit. The best-fit ARIMA models were expected to exhibit a low Akaike's information criterion (AIC), a low mean absolute error (MAE), a large coefficient of determination (*R*^2^), statistically significant (at the 5% level) regression coefficients for each meteorological parameter and ARIMA component, and a low variance inflation factor (VIF) among any multiple independent predictors. The goodness of fit was further confirmed through normality tests and autocorrelation analysis of model residuals. All statistical analyses and model developments were conducted using JMP 9.0 at the two-tailed significance level of *P* < 0.05 (or the 95% confidence level).

Independent evaluation of the best-fit models was performed using the 2011 verification data. The first few forecast periods for 2011 used developmental data from 2010, but later periods used only verification data with the predicted D_POP_ used for autoregression. Agreement between model predictions of D_POP_ (with lagged meteorological parameters) and the actual reported D_POP_ during 2011 was evaluated through MAE and confidence interval analyses.

## RESULTS

### Intraseasonal prediction: biweekly data.

Dengue incidence rates in Cali do not exhibit strong seasonal or annual cycles, but when outbreaks occur, incidence rates have a tendency to increase during the latter one-half of a warm-dry season, when the diurnal temperature range is large, and then decrease as the subsequent cooler-wet season begins (Figures 3–5). Cross-correlation analysis showed that several evaluated weather parameters were significantly associated with D_POP_ at various biweekly lags, but the most significant associations (within each meteorological group from Table 1) were for T_AVG_, ΔT_AVG_, and RH_AVG_ at a 1-biweek lag and RR_TOT_ at a 2-biweek lag (Table 2). Only these four lagged parameters were evaluated as potential covariate predictors in the subsequent model development; the others were excluded because of either large colinearity within their group (G32_DAYS_ with T_AVG_) (Table 3) or their most significant lag being more interseasonal (L18_DAYS_ at the biweekly equivalent of 3–4 months).

Initial exploratory tests showed that the general ARIMA form of AR(2) with no intercept regularly produced the best-fit intraseasonal model. Such form implies a strong autoregressive component to the D_POP_ time series with significant variability at monthly intervals. Thus, to provide a baseline before the inclusion of independent meteorological predictors, an AR(2) model fitted from only D_POP_ (hereafter model 1) is provided. The performance statistics for model 1 are shown in Table 4, and the full prediction equation is

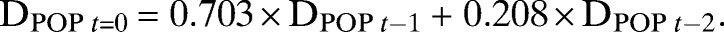


**Figure 7.**
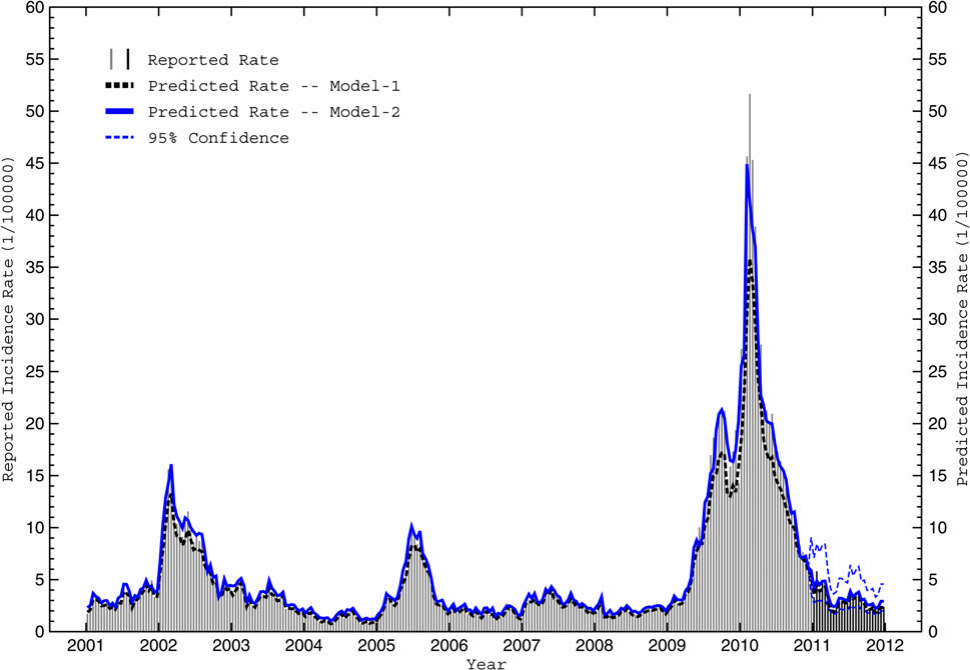
Reported and predicted intraseasonal dengue incidence rates from 2001 to 2011. The reported population-adjusted biweekly dengue incidence rates (D_POP_) for Cali during the model development period (2001–2010) are shown by vertical grey lines, whereas reported rates during the model verification period (2011) are denoted by vertical black lines. Also shown are biweekly predictions by the best intraseasonal autoregressive model not using weather parameters (model 1; dashed black) and the best model using lagged weather parameters (model 2; solid blue) during both subperiods. The dashed blue lines denote the 95% confidence interval for model 2 during the verification period.

Figure 7 shows the reported and model 1-predicted D_POP_ during the development (2001–2010) period. Overall, the model performed well (*R*^2^ = 0.884) but significantly underpredicted dengue incidence just before and during the three most severe outbreaks. To determine whether the inclusion of independent meteorological predictors could improve the prediction, all possible combinations of the four previously identified potential predictors were fitted with an AR(2) model using transfer functions (a total of 24 models) and evaluated. Performance statistics are shown in Table 4 for the best model (hereafter, model 2), which incorporates only ΔT_AVG_ information based on an initial 1-biweek lag. The full prediction (transfer) equation is

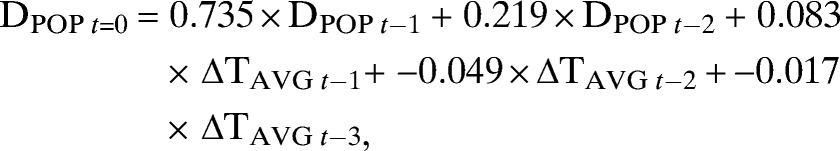


where the two additional apparent lags for ΔT_AVG_ (at *t* – 2 and *t* – 3) result from expanding the polynomials associated with transferring the second-order autoregressive backshift operator from the D_POP_ time series to the ΔT_AVG_ time series. Comparison of the regression coefficients (β) for ΔT_AVG_ and the cross-correlations coefficients in Table 3 suggests that dengue incidence rates increase soon after warm-dry periods when daily extreme temperatures are above average (e.g., ΔT_AVG_ is negatively associated with RR_TOT_, RH_AVG_, and T_MIN_ but positively associated with T_AVG_, L18_DAYS_, and G32_DAYS_). Model 2 satisfies all performance expectations (lower AIC, lower MAE, larger *R*^2^, and significant β), and the remaining residuals fluctuated randomly around zero with no significant trend or autocorrelation, further implying a well-fitted model. Conversion of the MAE for the predicted normalized D_POP_ to dimensional form results in an equivalent MAE of 1.04 dengue cases per 100,000 individuals.

Figure 7 shows the reported and model 2-predicted D_POP_ during the development (2001–2010) and verification (2011) periods. Incidence rates during the development period compare well (*R*^2^ = 0.928) with a 5–6% improvement in total variance explained compared with the model 1 baseline. Model 2 also captured the timing and magnitude the three severe outbreaks better than model 1 without losing predictive skill during the intermediate low-magnitude periods. Thus, although the overall statistical improvement may be modest, inclusion of meteorological information into the intraseasonal model improves its predictive skill for the most critical situations. Finally, during the independent verification period, all 26 biweekly predictions fell within the 95% confidence interval (*R*^2^ = 0.885), timing of the modest mid-year peak was well-captured, and the equivalent (dimensional) MAE was 0.61 dengue cases per 100,000 individuals.

### Interseasonal prediction: monthly data.

Cali's dengue incidence rates also exhibit relationships to the local climate and ENSO (Figures 3–6); the most severe outbreaks tend to occur over multiple seasons during El Niño events, when the mean rainfall is below average and temperatures remain warmer than 18°C. For example, the largest dengue outbreak (from late 2009 to early 2010) coincided with an exceptionally warm and dry 9-month period during a strong El Niño when the late year wet season failed and daily minimum temperatures never dropped below 17°C. Cross-correlation analysis showed that the most significant associations with D_POP_ were for T_AVG_, G32_DAYS_, and ΔT_AVG_ at a 1-month lag, L18_DAYS_ and RR_TOT_ at a 4-month lag, and RH_AVG_ and N_4_ at a 6-month lag (Table 5). Only the lagged L18_DAYS_, RH_AVG_, RR_TOT_, and N_4_ were included in the interseasonal model development as potential predictors; the lagged T_AVG_, G32_DAYS_, and ΔT_AVG_ were deemed equivalent to their biweekly counterparts and considered more intraseasonal.

The general AR(1) form with no intercept regularly produced the best-fit interseasonal model, further implying a strong autoregressive monthly component to D_POP_ prediction. Again, before the inclusion of independent meteorological predictors, an AR(1) model fitted from only D_POP_ (hereafter model 3) is provided as a baseline. The performance statistics for model 3 are shown in Table 6, and the full prediction equation is

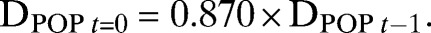


**Figure 8.**
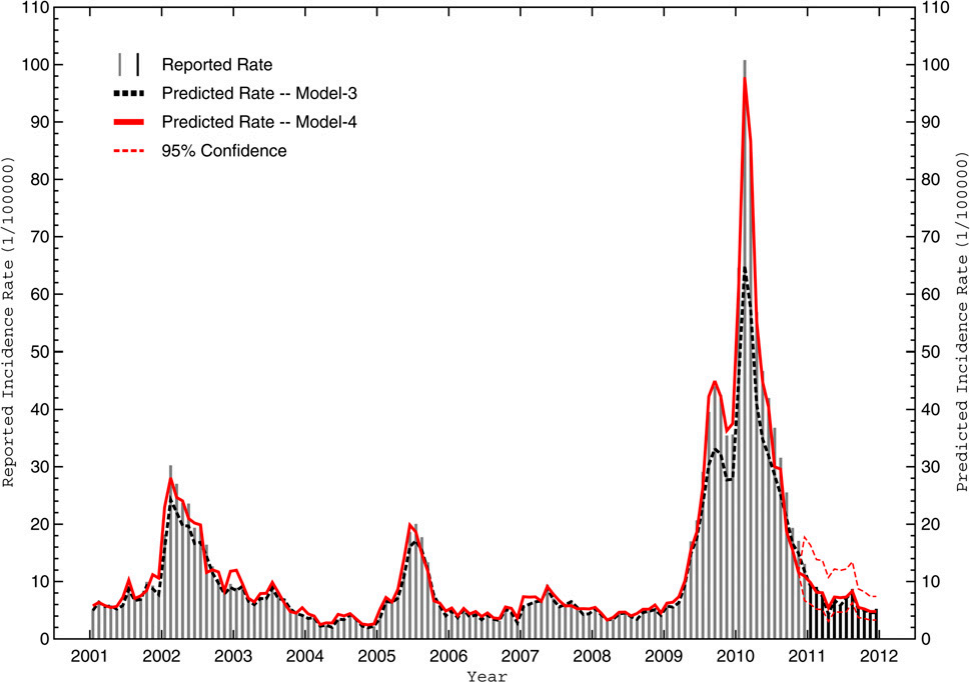
Reported and predicted interseasonal dengue incidence rates from 2001 to 2011. The reported population-adjusted monthly dengue incidence rates (D_POP_) for Cali during the model development period (2001–2010) are shown by vertical light grey lines, whereas reported rates during the model verification period (2011) are denoted by vertical medium grey lines. Also shown are monthly predictions by the best interseasonal autoregressive model not using weather parameters (model 3; dashed dark grey) and the best model using lagged weather parameters (model 4; solid black) during both subperiods. The thin dashed black lines denote the 95% confidence interval for model 4 during the verification period.

Figure 8 shows the reported and model 3-predicted D_POP_ during the development (2001–2010) period. Overall, the model performed well (*R*^2^ = 0.846), but again, it significantly underpredicted dengue incidence just before and during the three most severe outbreaks. Next, all possible combinations of the potential interseasonal meteorological predictors were fitted with an AR(1) model using transfer functions (24 total models) and evaluated. Performance statistics are shown in Table 6 for the best model (hereafter model 4), which incorporates RH_AVG_, L18_DAYS_, and N_4_ information based on initial 4- to 6-month lags. The full prediction (transfer) equation is

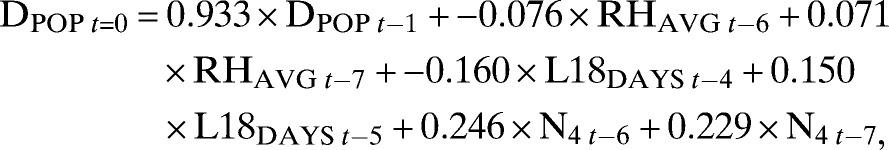


where like before, the additional apparent lags for RH_AVG_, L18_DAYS_, and N_4_ (at *t* − 5 and *t* − 7) result from expanding the polynomials associated with transferring the first-order autoregressive backshift operator from the D_POP_ time series to the meteorological time series. Comparison of the regression coefficients (β) and the cross-correlations coefficients in Table 3 supports the notion that dengue incidence rates increase in those months after a warm period that was preceded by an abnormally dry season coincident with peak El Niño conditions. Model 4 satisfied all performance expectations (lower AIC, lower MAE, larger *R*^2^, and significant β), and the remaining residuals exhibited no significant trend or autocorrelation. The model's equivalent (dimensional) MAE was 2.8 dengue cases per 100,000 individuals.

Figure 8 shows the reported and model 4-predicted D_POP_ during the development and verification periods. Incidence rates during the development period compare well (*R*^2^ = 0.901), with a 5.5% improvement in total variance explained compared with the model 3 baseline, and model 4 captured the severe outbreaks better than model 3 without losing skill during intermediate periods. Thus, as with the best intraseasonal model, inclusion of meteorological information into the interseasonal model improves its predictive skill for the most critical situations. Finally, during the verification period, all monthly predictions fell within the 95% confidence interval (*R*^2^ = 0.882), timing of the intra-annual extremes was well-captured, and the equivalent (dimensional) MAE was 1.4 dengue cases per 100,000 individuals.

## Discussion

The influence of meteorological factors on dengue transmission dynamics in Cali, Colombia during 2000–2010 has been established at biweekly and monthly time scales. Significant cross-correlation maxima between D_POP_ and various local weather and regional climate parameters were identified; positive associations were found for T_AVG_ (at a time lag of 1–2 weeks), G32_DAYS_ (1–2 weeks), ΔT_AVG_ (1–2 weeks), RR_TOT_ (4 months), and N_4_ SSTs (6 months), whereas negative associations were found for L18_DAYS_ (3–4 months), RH_AVG_ (1–2 weeks and 6 months), and RR_TOT_ (3–4 weeks). Using these intra- and interseasonal relationships, multivariate autoregressive models that best predict D_POP_ were developed for each timescale; the best intraseasonal (interseasonal) model used ΔT_AVG_ at a 1- to 2-week lag (used L18_DAYS_ at a 4-month lag and RH_AVG_ and N_4_ at 6-month lags). Both models showed precision (high *R*^2^) and skill (low MAE) during the 2000–2010 development and 2011 verification periods. Moreover, inclusion of meteorological predictors into both autoregressive models provided significant forecast skill improvement during the most critical situations (during severe dengue outbreaks) compared with their autoregressive counterparts without meteorological predictors.

### Influence of local weather variability on dengue dynamics.

Based on our time series analysis, variability in intraseasonal weather plays a significant role in dengue transmission across Cali, Colombia; reported cases often peaked 2–4 weeks after a warm-dry period when ΔT_AVG_ was above average. Such prior environmental conditions are broadly consistent with other weather-based EWSs for dengue.^13^,^27^,^36^,^39^ Moreover, a recent study discussed the expected time lag between mosquito birth and the appearance of clinical symptoms in humans: larval and pupa development (10–21 days), a gonotrophic cycle (3–7 days per cycle), extrinsic incubation in mosquitoes (7–15 days), and in-human incubation (1–12 days) for a total lag of 21–55 days (or 3–8 weeks).^39^ Because the rate of these sequential events increases at warmer temperatures^20^,^22^ and feeding frequency increases with lower humidity,^24^ our findings that the number of dengue cases often peaks 2–4 weeks after a warm-dry period are consistent with such expected lags.

Our most unexpected finding was that lagged ΔT_AVG_ provided the best prediction of dengue incidence. This study is the first empirical study that specifically evaluated the relationships between ΔT_AVG_ and dengue incidence. A recent study, through a combination of laboratory experiments and a thermodynamic model, showed that dengue transmission could be highly dependent on ΔT_AVG_.^32^ In particular, it showed that, at mean temperatures less than (greater than) 18°C, larger ΔT_AVG_ resulted in increased (reduced) transmission because of increases (reductions) in viral amplification and vector survival during the warmer (colder) hours of each day. Hence, the study suggests that maximum dengue transmission would occur for those combinations of T_AVG_ and ΔT_AVG_ that minimize the number of daily hours in which ambient temperatures are outside the optimal 18–32°C range for mosquito survival and dengue transmission.^20^ Figures 4–6 illustrate the number of days with non-optimal temperature extremes (L18_DAYS_, G32_DAYS_, and similar parameters computed over small ranges) for Cali during 2000–2011. Note how each significant dengue outbreak was preceded by multiple weeks (or months) when extreme daily temperatures were largely confined within the optimal 18–32°C range. The relationship is strongest with the minimum temperatures (Figure 4), suggesting that prolonged warm periods (whereby temperatures rarely drop below 18°C) foster vector population growth, faster virus amplification in the larger population, and increased vector feeding and human host contact, leading to increases in human infections.^22^ In contrast, cooler periods (when temperatures often drop below 18°C) provide a regular effective brake on vector population growth and viral development, which reduces human host contact and limits the number of infections.

Why then was lagged ΔT_AVG_ our strongest local predictor of dengue incidence for Cali if the frequency of extreme temperatures seems more critical for vector survival and dengue transmission? This finding may result from considerable overlap among the optimal temperature range for transmission (18–32°C), the climatological range for extreme daily temperatures in Cali (15–35°C), the variability in this overlap on intraseasonal time scales, and its relation to other meteorological parameters (RH_AVG_ and RR_TOT_) that influence vector populations and disease transmission. Indeed, inspection of Table 3 reveals that ΔT_AVG_ is the only parameter to exhibit statistically significant relationships with each parameter used in previously developed weather-based EWSs for dengue (T_AVG_, T_MIN_, T_MAX_, RH_AVG_, and RR_TOT_)^13^,^27^,^33^,^36^,^39^ while also exhibiting significant associations with the metrics for non-optimal temperatures (L18_DAYS_ and G32_DAYS_). In other words, variability in ΔT_AVG_ alone provides a holistic measure of the aforementioned environmental conditions known to be favorable for dengue transmission.

However, why were lagged RH_AVG_ and RR_TOT_ not significant predictors of intraseasonal dengue incidence for Cali? The cross-correlation analysis with D_POP_ (Table 2) implied that dengue incidence peaked after dry periods initiated up to 6 weeks before. Similar lagged relationships were found for Singapore^27^ and New Caledonia,^36^ but other tropical locales exhibited either increased dengue incidence after wet periods^13^,^33^,^34^,^37^ or no correlation.^21^ Two plausible explanations are suggested. First, moist tropical regions (such as Cali) may exhibit weaker correlations between rainfall variability and mosquito density than dry tropical or subtropical regions (such as Taiwan) if the moist regions receive sufficient rainfall to maintain a regular supply of abundant breeding sites.^9^,^29^ Second, variability in rainfall intensity may be more critical than total accumulation, because heavy rainfall can destroy larvae and reduce the survival rate of adult female mosquitoes, whereas light rainfall increases the usage of water storage containers that may serve as breeding sites.^27^,^28^ Resolution of this speculation through detailed multisite analysis is left for future work.

### Influence of regional climate variability on dengue dynamics.

Our findings also support the hypothesis that interseasonal climate variability, linked to ENSO, plays an important role in dengue transmission across Cali; incidence often peaked 4–6 months after an El Niño event associated with above-average local temperatures and below-average local rainfall and humidity. Similar ENSO-lagged relationships were found for Mexico^50^ and Thailand,^51^ although the relationships seem to be complex and exhibit signs of non-stationarity.^52^,^53^

How does ENSO impact local Cali weather? The (objective) statistical selection of N_4_ as the best predictor among the five ENSO indices is consistent with the traditional notion that the N_4_ index is best correlated with the eastward shift of the Walker Circulation during an El Niño event.^54^ This eastward shift often enhances subsidence (and suppresses convection) over Colombia. At the same time, the SST gradient between the Colombian coast and the normally cold waters off Peru and Ecuador decreases, weakening the easterly winds and moisture advection (from the Amazon) into central/western Colombia and further lowering humidity and limiting rainfall.^55^ Indeed, during 1980–1999, the Colombian climate in years immediately after an El Niño event was characterized by less rainfall, lower humidity, warmer temperatures, and a national increase in dengue incidence.^56^ Our 2000–2010 interseasonal cross-correlation analysis with D_POP_ (Figures 6 and 8 and Table 5) is broadly consistent with such a local response to remote ENSO forcing.

The interseasonal impacts of ENSO on local dengue dynamics are probably most facilitated through its subsidence-induced drying effects, which in turn, could enhance the long-term abundance of water storage containers for use as breeding sites. Because *Ae. aegypti* eggs, under laboratory conditions, can withstand desiccation for up to 4 months depending on ambient humidity levels,^57^ it seems plausible that the lagged RH_AVG_ predictor (at 6 months) reflects an interseasonal memory, whereby moisture conditions during prior wet/dry seasons influence the number of dengue cases in the current season. Similar interseasonal lags between dengue incidence and either moisture or rainfall have been noted.^35^,^38^,^58^

### Influence of other factors.

Although our results show that weather and climate have played significant roles in facilitating recent dengue transmission across Cali, transmission dynamics are a function of complex relationships between hosts, vectors, and their environment. Several other confounding factors may contribute to the observed intra- and interseasonal variability of dengue incidence. First, Cali has experienced rapid population growth over the past two decades (∼35% since 1993), primarily in the lower socioeconomic neighborhoods, where simple vector control measures are less common, housing density is greater, underreporting is more common, and water storage containers are more abundant.^43^,^44^,^59^ Second, budget constraints have reduced timely vector control programs, and the local *Ae. aegypti* populations has exhibited at least partial resistance to all commercially available insecticides.^3^,^60^,^61^ Third, given the close proximity of Colombia to the equatorial tropical Pacific, we only evaluated ENSO–dengue relationships, but other modes of climate variability, such as the Madden–Julian Oscillation (a 30- to 90-day cycle)^62^ and the North Atlantic Oscillation (a 3- to 5-year cycle),^63^ may play significant interseasonal roles. Fourth, ecological factors, such as seasonal vegetation dynamics,^58^ may influence vector density on the local scale. Finally, interannual variability in dengue incidence can be related to the rotation of herd immunity (by human hosts) through the four serotypes.^19^,^36^,^39^

In our predictive models, such confounding factors are collectively represented through the autoregressive D_POP_ terms. Moreover, the large AR regression coefficients (Tables 4 and 6) imply that the density of infected hosts remains a strong short-term predictor of subsequent incidence rate. Thus, until the full range of factors influencing dengue transmission is known, the use of autoregressive models in local EWSs seems to be an effective means to incorporate confounding factors that are unique to a given region.^27^,^33^,^37^,^39^

### Integration of weather-based prediction into multicomponent EWSs.

Until an effective vaccine or antiviral drug for dengue fever becomes available, EWSs will remain an essential tool for curbing dengue transmission and reducing the case numbers. However, the implementation of an EWS can be resource- and labor-intensive, posing an economic burden on communities with limited resources. Given the complex dynamics of dengue transmission, an effective low-cost EWS requires a multicomponent approach that combines (1) environmental surveillance, including weather-based predictive models, (2) entomological surveillance, (3) proactive vector control strategies, including the targeted use of insecticides and breeding habitat reduction, (4) public awareness and education, and (5) timely emergency response and case management.

In Colombia, dengue outbreaks were managed historically through vector eradication efforts using widespread dichloro-diphenyl-tricloroethane (DDT) spraying, but the *Ae. aegypti* became highly resistant to the insecticide.^3^,^60^,^61^ Since the 1980s, vector control strategies have followed an integrated community-based approach, combining limited entomological surveillance and regular application of chemical and bacterial larvicides to permanent stagnant water sources; insecticide spraying is used only after dengue outbreaks are confined.^3^,^64^,^65^

The predictive models described herein could be integrated into a low-cost dengue EWS for Cali. Both models use publicly available weather and climate data, which remove any need for financial investment in weather-based predictive methods. The interseasonal model could be used to initiate proactive public awareness and education campaigns as well as long-term resource planning, such as setting municipal budgets and acquiring sufficient mitigation resources. The intraseasonal model could then be used to guide any initial outbreak response efforts, such as the acquisition of sufficient medical supplies, timely public awareness reminders, and intensity of targeted vector control efforts. Studies have shown that such low-cost weather-based EWSs can help mitigate potential dengue epidemics in a timely manner.^34^–^36^,^38^

### Conclusions and future work.

A comprehensive global dengue research agenda requires simultaneously addressing known deficiencies in the medical, public, and health policy, vector control, and vector surveillance arenas.^66^ Here, we contribute to the latter by developing weather-based intra- and interseasonal prediction models that can be integrated into the existing community-based EWSs for Cali, Colombia and provide sufficient lead time to initiate effective vector control and medical response operations when periods of elevated dengue risk are predicted.

Our results also suggest new avenues for future study. First, it seems important to further evaluate the impact of daily temperature variability on the behavior and full lifecycle of the *Ae. aegypti* mosquito, because significant dengue outbreaks often occurred when extreme daily temperatures are confined within the 18–32°C range. Second, more research is needed to develop spatiotemporal predictive models of dengue fever incidence. Patterns of spatial variability across endemic regions (such as Colombia) may be related to variations in the built environment, ecology, local weather and climate, population density/migration, mitigation efforts, and host mobility. A few recent studies have evaluated spatial dengue transmission pattern, but unique site-specific factors limit the extrapolation of their results to other geographic regions.^4^,^43^,^59^,^67^ Given that global climate change is expected to spread the risk of dengue fever into higher latitudes and higher elevations and to a greater percentage of the global population,^9^,^11^,^16^ such efforts could provide effective regional EWSs for all at-risk populations.

## Figures and Tables

**Table 1:** Weather and climate parameters evaluated for use in the predictive models

Weather/climate parameter	Symbol	Category	Biweekly	Monthly
Maximum temperature (°C)	T_MAX_	Temperature	X	X
Mean temperature (°C)	T_AVG_	Temperature	X	X
Minimum temperature (°C)	T_MIN_	Temperature	X	X
Number of days with T_MIN_ < 18°C	L18_DAYS_	Temperature	X	X
Number of days with T_MAX_ > 32°C	G32_DAYS_	Temperature	X	X
Mean daily temperature range (°C)	ΔT_AVG_	Temperature range	X	X
Maximum daily temperature range (°C)	ΔT_MAX_	Temperature range	X	X
Mean relative humidity (%)	RH_AVG_	Humidity	X	X
Relative humidity range (%)	ΔRH	Humidity	X	X
Total rainfall (mm)	RR_TOT_	Rainfall	X	X
Number of days with measurable rainfall	RR_DAYS_	Rainfall	X	X
SOI	SOI	Climate		X
N_12_ index	N_12_	Climate		X
N_3_ index	N_3_	Climate		X
N_4_ index	N_4_	Climate		X
N_34_ index	N_34_	Climate		X

**Table 2:** Lagged cross-correlation coefficients between selected biweekly weather parameters and the population-adjusted dengue fever incidence rate (D_POP_) during the development period from 2000 to 2010

Lag (biweekly)	T_AVG_	L18_DAYS_	G32_DAYS_	ΔT_AVG_	RH_AVG_	RR_TOT_
−8	0.086	−0.274*	0.091	−0.073	−0.006	0.025
−7	0.122	−0.330*†	0.098	−0.078	−0.090	0.019
−6	0.092	−0.261*	0.113	0.072	−0.028	−0.043
−5	0.121	−0.173*	0.113	−0.005	−0.091	−0.082
−4	0.136*	−0.161*	0.126*	−0.016	−0.005	−0.111
−3	0.137*	−0.129*	0.082	0.166*	−0.047	−0.123*
−2	0.249*	−0.093	0.207*	0.305*	−0.155*	−0.136*†
−1	0.305*†	−0.073	0.241*†	0.328*†	−0.314*†	−0.124*
0	0.255*	−0.057	0.213*	0.253*	−0.264*	−0.053

* Statistical significance at the 5% level after accounting for serial correlation and pre-whitening the time series.

† Maximum coefficients.

**Table 3:** Cross-correlation coefficients at zero lag among selected monthly weather and climate parameters during the development period from 2000 to 2010

Parameters	T_AVG_	ΔT_AVG_	RH_AVG_	RR_TOT_	T_MIN_	L18_DAYS_	T_MAX_	G32_DAYS_	N_4_	N_34_
T_AVG_	−	−	−	−	−	−	−	−	−	−
ΔT_AVG_	0.55*	−	−	−	−	−	−	−	−	−
RH_AVG_	−0.76*	−0.63*	−	−	−	−	−	−	−	−
RR_TOT_	−0.51*	−0.39*	0.53*	−	−	−	−	−	−	−
T_MIN_	0.17	−0.42*	0.08	0.03	−	−	−	−	−	−
L18_DAYS_	−0.20	0.50*	−0.08	−0.05	−0.79*	−	−	−	−	−
T_MAX_	0.66*	0.72*	−0.64*	−0.29	−0.04	0.05	−	−	−	−
G32_DAYS_	0.77*	0.69*	−0.58*	−0.41*	0.02	0.02	0.87*	−	−	−
N_4_	0.58*	0.32	−0.30	−0.35*	0.02	−0.24	0.38*	0.60*	−	−
N_34_	0.61*	0.30	−0.32	−0.32	0.07	−0.27	0.37*	0.61*	0.94*	−

The cross-correlations coefficients for the same set of biweekly parameters (excluding N_4_ and N_34_) are not shown, because they exhibit similar magnitudes, signs, and levels of statistical significance.

* Statistical significance (from zero) at the 5% level.

**Table 4:** Summary statistics for the best intraseasonal autoregressive prediction models of dengue incidence without using lagged weather parameters (model 1) and using lagged weather parameters (model 2)

Variable	Lag	β	SE	*R*^2^	MAE	AIC	VIF
Model 1				0.884	0.220	134	1.00
D_POP_	AR1	0.703*	0.057				
D_POP_	AR2	0.208*	0.057				
Model 2				0.928	0.216	131	1.00
ΔT_AVG_	−1	0.083*	0.022				
D_POP_	AR1	0.735*	0.059				
D_POP_	AR2	0.219*	0.059				

The full prediction equations are provided in the text.

* Statistical significance (from zero) at the 5% level.

**Table 5:** Lagged cross-correlation coefficients between selected monthly weather/climate parameters and the population-adjusted dengue fever incidence rate (D_POP_) during the development period from 2000 to 2010

Lag (monthly)	T_AVG_	L18_DAYS_	G32_DAYS_	ΔT_AVG_	RH_AVG_	RR_TOT_	N_4_
−8	0.128	−0.005	0.003	0.046	−0.115	−0.018	0.100
−7	0.224*	0.072	0.145*	0.213*	−0.183*	0.008	0.163*
−6	0.219*	0.033	0.185*	0.117	−0.360*†	0.046	0.252*†
−5	0.205*	−0.158*	0.161*	0.064	−0.246*	0.113	0.143*
−4	0.179*	−0.315*†	0.055	−0.006	−0.138*	0.142*†	0.047
−3	0.224*	−0.241*	−0.046	0.059	−0.130	0.059	0.101
−2	0.264*	−0.096	0.023	0.142*	−0.048	−0.113	0.218*
−1	0.393*†	−0.008	0.274*†	0.320*†	−0.251*	−0.132*	0.193*
0	0.159*	−0.039	0.219*	0.197*	−0.170*	−0.097	0.059

* Statistical significance at the 5% level after accounting for serial correlation and pre-whitening the time series.

† Maximum coefficients.

**Table 6:** Summary statistics for the best interseasonal autoregressive prediction models of dengue incidence without using lagged weather/climate parameters (model 3) and using lagged weather/climate parameters (model 4)

Variable	Lag	β	SE	*R*^2^	MAE	AIC	VIF
Model 3				0.846	0.251	92	1.00
D_POP_	AR1	0.870*	0.029				
Model 4				0.901	0.229	76	1.18
RH_AVG_	−6	−0.076*	0.030				
L18_DAYS_	−4	−0.161*	0.052				
N_4_	−6	0.246*	0.089				
D_POP_	AR1	0.932*	0.029				

The full prediction equations are provided in the text.

* Statistical significance (from zero) at the 5% level.
